# Trends in Antibiotic Prescribing in Adults in Dutch General Practice

**DOI:** 10.1371/journal.pone.0051860

**Published:** 2012-12-12

**Authors:** Michiel B. Haeseker, Nicole H. T. M. Dukers-Muijrers, Christian J. P. A. Hoebe, Cathrien A. Bruggeman, Jochen W. L. Cals, Annelies Verbon

**Affiliations:** 1 Department of Medical Microbiology, Maastricht University Medical Centre, Maastricht, The Netherlands; 2 Department of Sexual Health, Infectious Diseases, and Environmental Health, Public Health Service South Limburg, Geleen, The Netherlands; 3 Department of General Practice, Maastricht University, Maastricht, The Netherlands; 4 School for Public Health and Primary Care (CAPHRI), Maastricht, The Netherlands; 5 Department of Internal Medicine Section, Infectious Disease, Erasmus Medical Centre, Rotterdam, The Netherlands; University of Ottawa, Canada

## Abstract

**Background:**

Antibiotic consumption is associated with adverse drug events (ADE) and increasing antibiotic resistance. Detailed information of antibiotic prescribing in different age categories is scarce, but necessary to develop strategies for prudent antibiotic use. The aim of this study was to determine the antibiotic prescriptions of different antibiotic classes in general practice in relation to age.

**Methodology:**

Retrospective study of 22 rural and urban general practices from the Dutch Registration Network Family Practices (RNH). Antibiotic prescribing data were extracted from the RNH database from 2000–2009. Trends over time in antibiotic prescriptions were assessed with multivariate logistic regression including interaction terms with age. Registered ADEs as a result of antibiotic prescriptions were also analyzed.

**Principal Findings:**

In total 658,940 patients years were analyzed. In 11.5% (n = 75,796) of the patient years at least one antibiotic was prescribed. Antibiotic prescriptions increased for all age categories during 2000–2009, but the increase in elderly patients (>80 years) was most prominent. In 2000 9% of the patients >80 years was prescribed at least one antibiotic to 22% in 2009 (*P*<0.001). Elderly patients had more ADEs with antibiotics and co-medication was identified as the only independent determinant for ADEs.

**Conclusion/Discussion:**

The rate of antibiotic prescribing for patients who made a visit to the GP is increasing in the Netherlands with the most evident increase in the elderly patients. This may lead to more ADEs, which might lead to higher consumption of health care and more antibiotic resistance.

## Introduction

The majority of antibiotics (80%) in the Netherlands are prescribed in primary care [1]. Outpatient antibiotic consumption is higher in elderly patients than in the general population [2]–[4] and most antibiotics are prescribed in elderly patients for respiratory tract infections (RTI) [5], skin and soft tissue infections [6] and urinary tract infections (UTI) [7]. However, detailed information of antibiotic prescribing in elderly is scarce. The majority of studies have been done in children, who also have a high antibiotic consumption [2], [8], [9]. The paucity of data on antibiotic use in elderly is surprising since elderly patients are more susceptible to toxic effects of antibiotics. For instance, adverse drug events (ADEs) have been described more frequently in frail elderly with co-morbidity and co-medication [10]. Additionally, elderly patients have altered pharmacokinetics, such as decreased absorption and elimination, which alters antibiotic blood levels, thereby influencing the risk of ADEs [11].

Antibiotic use is slowly, but steadily increasing in the Netherlands since 2005 [12]. It is unknown whether the increase in antibiotic use is equal in all age categories. Trends over time in antibiotic use per age category have not been studied and more information on the highest age categories is crucial as a quarter of the Dutch population will be above 65 years in the near future, similar to other European countries [13].

To determine trends in antibiotic prescribing in elderly, we have assessed antibiotic prescription rates by age categories in general practices over a ten year period in a large general practice database. Additionally, we have analyzed the incidence of registered ADEs due to antibiotics.

## Methods

The data for this study are obtained retrospectively from the Dutch Registratie Netwerk Huisartsen (RNH, Registration Network Family Practices). The study group is described and regrouped into four different age categories: 18–44 years, 45–64 years, 65–79 years and ≥80 years. In these age categories, we have compared rates and trends over time in antibiotic prescriptions. Individual prescriptions per patient in a year were used as measure of prescriptions. For each prescription (at the moment of the specific consultation) information on ADE (until 4 weeks after prescription) and co-medication (at and during the prescription) were used. Data were then aggregated over a specific patient and calendar year to obtain a meaningful measure (% per patient-year).

### Data Source

The RNH is a continuous, computerized and anonymous database from 22 rural and urban general practices in the south of the Netherlands, Limburg, [14]. During the study period the average number of patients >18 years in the RNH was 65,894 patients. The population was stable with respect to general sociodemographic characteristics.

The GP is responsible for the inclusion of patients. When a patient is included a unique RNH number is attributed to the patient. The GP records patient characteristics, i.e. birthdates, sex, educational level, insurance, type of household, marital status, place of residence, date of entry, last update and end of registration. The GP records all relevant health problems, only permanent, chronic and recurrent (>3 recurrences within a period of 6 months) are recorded, or when they had lasting consequences for the functional status or prognosis of the patient. Medication prescriptions (coded according to the Anatomical Therapeutic Chemical Index 2012 by the WHO Collaborating Centre for Drug Statistics Methodology) are included in the RNH database Registration. This includes type of medication, start and end date, and dose. Diagnoses are coded in a standardized fashion, according to the International Classification of Primary Care, using the criteria of the International Classification of Health in Primary Care and the current guidelines of the Dutch College of GPs [15]. Monitoring by the RNH registry ends at migration or death. All practices use MicroHIS (Torex-Hiscom), a commercially developed general practice health information software program containing a basic module, a medical module and a pharmacy module, which enable the GP to keep up an automated registration of his patients. Quarterly the RNH data collection module enables the GP to exchange all registered data to the central database. The RNH assistant checks the data and the RNH test module provides the distribution of the practice population, e.g. tables with the age distribution and the twenty most registered ICPC codes. The RNH exports the database in an SPSS data format.

A number of instruments are available in order to improve the quality and to reduce the inter-doctor variation: all GPs participating in the RNH are instructed and trained, RNH help program exists with all guidelines and criteria described, regional consensus group meeting take place at least 4 times a year to discus their registration difficulties, special software for data control in the health information system used by the GP and, special software for data control in the central database, several quality control experiments have been performed to gain insight into the quality of the database and several measures of agreement were done [14], [16].

### Data Analysis

Chronic diseases are selected from the classification from chronic diseases of Knottnerus [17]. We have included all chronic diseases, only congenital diseases have been excluded. Using ATC code J01 for all antibacterial medication for systemic use, further discrimination is made for antibiotic classes used in general practice, i.e. tetracycline (ATC J01A), penicillins (ATC J01C), sulphonamides and trimetroprim (ATC J01E), macrolides (ATC J01F), fluoroquinolones (ATC J01MA) and nitrofuran derivates (ATC J01XE). Antifungals (J02) and anti-tuberculosis drug (J03) excluded All other medication present at the moment when antibiotics have been prescribed are considered co-medication. Co-medication is registered in the following groups of medication: drugs for peptic ulcer and gastro-esophageal reflux (ATC A02B), antithrombotic agents (ATC B01A), cardiovascular medication (ATC C), corticosteroids for systemic use (ATC H02), anti-inflammatory and anti-rheumatic products, non-steroids anti-inflammatory drugs (ATC M01A), other analgesics and antipyretics (ATC N02B), anxiolytics (ATC N05B), hypnotics and sedatives (ATC N05C), antidepressants (ATC N06A), and antihistamines for systemic use (ATC R06). Since 1996 the RNH is recording all ADEs as ICPC code A85. All kinds of ADEs could be recorded at the discretion of the attending GP. ADEs are considered associated with antibiotic prescription if occurring within four weeks after start of antibiotics. The ADEs are self-reported by patients and were recorded as an ADE at the discretion of the attending GP.

### Statistical Analysis and Ethics

Unit of analyses are patient years, where a patient contributed one patient-year when he or she had attended a GP in that calendar year. Outcome measures included in the analyses are: antibiotic prescriptions (yes/no in a year), the number of antibiotic prescriptions (cumulated within a patient over a year) and ADEs (yes/no in a year). For antibiotic prescriptions, a multivariate model was build including age, time, gender, education level and rural versus and urban general practice, and chronic disease. In analyses with ADE as outcome restricted the study population to patient-years with antibiotic prescription and the multivariate model included age, time, gender, education level and rural versus and urban general practice, chronic disease and co-medication. The variables on age and time were presented, as these were our main focus, while controlling for the other variables. Interaction terms between age and time were explored. Statistical analysis is done with SPSS 16.0. A p-value <0.05 is considered statistically significant.

All patients included in the RNH database have been informed about the potential anonymous use of their health information. If a patient does not agree, the inclusion of this patient in the RNH database is stopped. All data in this study were analyzed anonymously, only medications and clinical data were used. The Medical Ethics Committee of the Maastricht University Medical Center approved this study (METC 12-4-053).

## Results

A total of 658,940 patient years were analyzed from 2000–2009: 43% (n = 280,417) of the patients years were aged between 18–44 years, 36% (n = 237,524) between 45–64 years, 16% (n = 108,131) between 65–79 years and 5.0% (n = 32,868) ≥80 years. Forty eight percent (n = 318,621) of the patient years were comprised by male and 52% (n = 340,319) were comprised by female, see Table 1 for population characteristics of the RNH. Sociodemographical characteristics of samples in other studies, which made use of the RNH database, have shown to be comparable to the Dutch population [16]. The elderly (>65 years) are slightly overrepresented in the RNH, 22% compared to 14% in the Dutch population in 2007. Hence, we do consider our results to be accurate and representative for the Dutch population, with a high internal and external generalizability.

**Table 1:** Population characteristics of the RNH in patient years.

	Antibiotic prescription		Total
	No	Yes	
Age categories			
• 18–44 yr	252,120 (43%)	28,297 (37%)	280,417 (43%)
• 45–64 yr	210,943 (36%)	26,581 (35%)	237,524 (36%)
• 65–79 yr	92,691 (16%)	15,440 (20%)	108,131 (16%)
• >80 yr	27,390 (5%)	5,478 (7%)	32,868 (5%)
Gender			
• Male	289,966 (50%)	28,655 (38%)	318,621 (48%)
• Female	293,178 (50%)	47,141 (62%)	340,319 (52%)
Education			
• Secondary school or lower secondary vocational education	188,361 (32%)	30,161 (40%)	218,522 (33%)
• Senior secondary vocational education	81,523 (14%)	11,905 (16%)	93,428 (14%)
• Higher education and University	26,248 (5%)	3,352 (4%)	29,600 (5%)
• Unknown	287,012 (49%)	30,378 (40%)	317,390 (48%)
Chronic disease			
• No	369,062 (63%)	39,161 (52%)	408,233 (62%)
• Yes	214,082 (37%)	36,635 (48%)	250,717 (38%)
General Practice			
• Rural	386,365 (66%)	45,900 (61%)	432,265 (66%)
• Urban	196,779 (34%)	29,896 (39%)	226,675 (34%)
Total	583,144 (85%)	75,796 (15%)	658,940 (100%)

**Figure 1:**
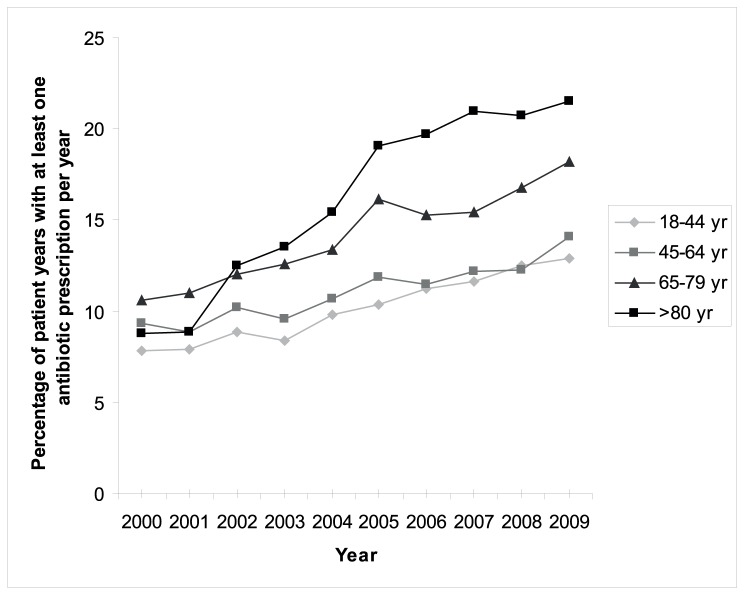
Percentage of patients years with at least one antibiotic prescription that year in different age categories during 2000–2009 (p<0.001).

**Figure 2:**
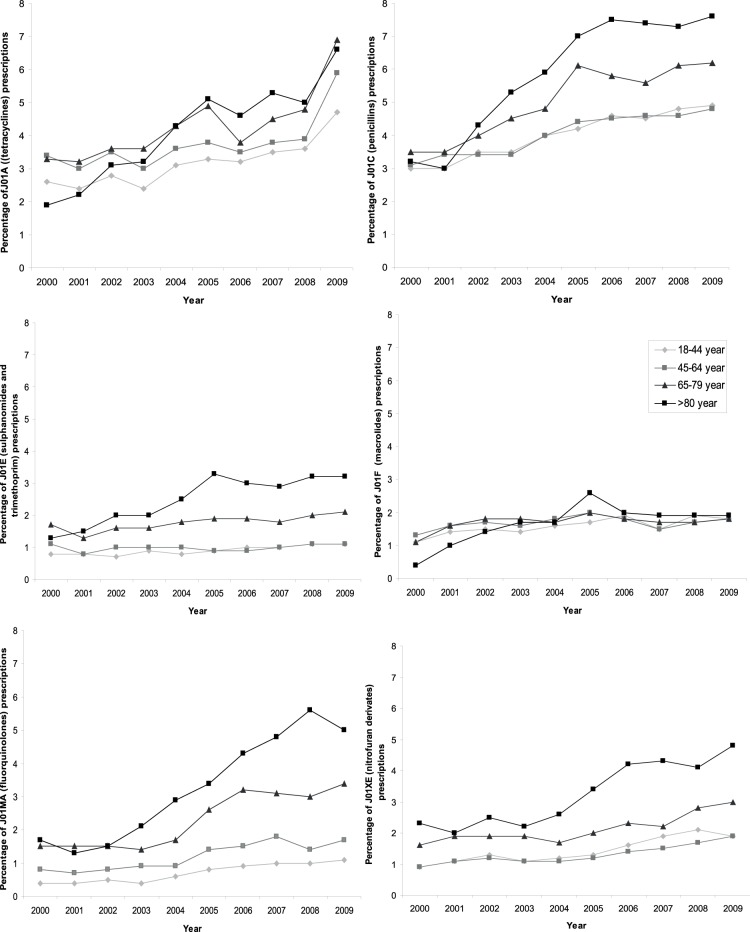
Percentage of patients with at least one antibiotic prescription that year for different classes of antibiotic prescriptions in different age categories during 2000–2009 (p<0.001).

In total 11.5% (n = 75,796) of the patient years at least one antibiotic per year was prescribed. Antibiotics were more often prescribed in elderly patients (p<0.001), as shown in **Figure 1**. The association with higher age was present in all years studied. Prescription rates of all antibiotics increased in all age-categories over time (all p<0.001). Although an increase was observed in all age categories, the increase was strongest in patients >80 years (interaction between age and time: p<0.001). This increase is seen for all antibiotic classes, with the exception of the macrolides (**Figure 2**). The number of antibiotic prescriptions per patient per year increased with age (p<0.001): two or more antibiotic courses were prescribed for 18% (n = 1,571) of the patients years in 18–44 years, 19% (n = 5,042) in 45–64 years, 23% (n = 3,581) in 65–79 years and 29% (n = 1571) in ≥80 years. The number of prescriptions independently increased with age and independently increased over time (p<0.001).

### ADEs, Underlying Diseases and co-medication

**Figure 3:**
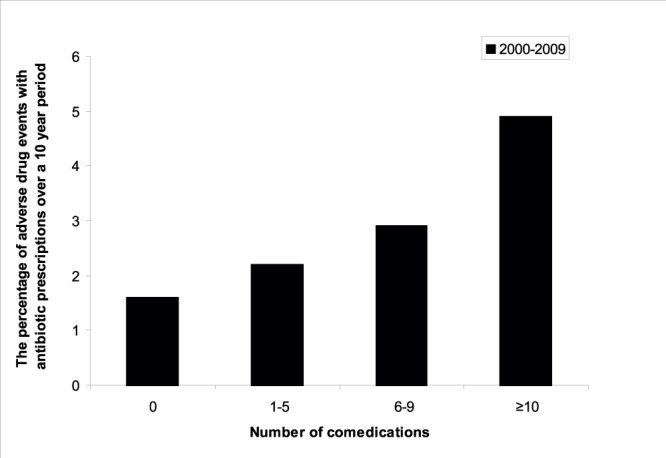
Percentage of adverse drug events with number of co-medication per age category during 2000–2009.

Of all patients who received an antibiotic prescription, the minority (2%; n = 1,526/75,796) reported an ADE in the four weeks time window. In a random sample of 4 week periods from the same patients, compared to any 4 week period, excluding antibiotic prescriptions during this period, rates found were 0.06% (n = 425/658,346) without antibiotics. No time trend in ADE reporting was observed. ADEs were not reported more frequently in the later years (2008/2009) compared to the earlier years (2000/2001). ADEs increased per age category per calendar year from 1.8% in 18–44 years, 1.9% in 45–64 years and 2.3% in 65–79 years to 2.8% in ≥80 years. However, co-medication also increased per age category from 23% (n = 6,529) in 18–44 years, 49% (n = 13,078) in 45–64 years and 70% (n = 10,978) in 65–79 years to 78% (n = 4,275) in ≥80 years. As expected, underlying chronic diseases increased by age. Of all patient years analyzed 18% (n = 50,924) were diagnosed with at least one chronic disease, 40% (n = 94,376) in the 45–64, 72% (n = 77,530) in 65–79 and 85% (n = 27,887) in >80 years age category. In the univariate analysis older age, having one or more chronic disease and co-medication were associated with occurrence of ADE. In the multivariate analysis use of co-medication remained associated with ADE (p<0.001) (**Figure 3**), while age and chronic disease were not.

## Discussion

This large primary cohort shows that the rate of antibiotic prescribing for patients who made a visit to the GP has increased in Dutch general practice from 2000 to 2009 in all adult age categories. We have observed the highest increase in the rate of antibiotic prescriptions in elderly patients; from 9% of patients receiving at least one antibiotic in 2000 to 22% in 2009. Additionally, elderly patients more often receive two or more antibiotic prescriptions per patient than younger patients. We have shown increasing trends for all antibiotic subgroups. This is particularly striking for the fluoroquinolones, as these are not indicated as first choice in any Dutch general practice guideline. In the United States nonapproved fluoroquinolone prescribing has already been described in 2005 [18]. Only the macrolides have shown a stable prescription pattern over time and across groups. Elderly patients have more ADEs associated with antibiotics. Co-medication has been identified as the only independent determinant for ADEs. Age was not in the multivariate analysis.

Our finding that the antibiotic prescription is increasing since 2005 in the Netherlands is comparable with the increase in Europe as shown in the European Surveillance of Antimicrobial Consumption data [19]. Although, the prescription rate in the Netherlands is increasing, it is still low compared to other European countries [19]. The Dutch guidelines for GPs of common infections have not been changed significantly during these ten-years. However, the increase of the number of fluoroquinolone prescriptions suggests that guidelines are not always followed. The increase in antibiotic prescriptions might be explained by an increased consultation frequency for acute infections, such as RTIs and UTIs. Although, the consultation frequency historically has tended to decrease over the years [20], it was recently shown that consultation rates for lower RTIs are increasing in the Netherlands while RTI related consultations are stable in the UK [21]. In this study however, we did not have access to the ICPC coding for acute conditions such as acute respiratory tract infections.

To our knowledge only three previous studies, one in England/Wales, one in New Zealand and one in Italy, have shown higher antibiotic consumption in elderly patients (>75 years) [2], [3]. We showed that elderly patients consistently have high antibiotic prescription rates compared to the younger patients and there is an increasing trend over time as well in these in elderly patients.

The highest risk group to develop ADEs is aged over 80 years with multiple co-morbidity and co-medication [22], [23]. Inappropriate prescriptions are a leading cause for the development of ADEs [24], [25]. Although, the observed rate of ADEs in our study is lower than the 5–35% found in other studies [26]–[28] our study is in line with studies that show that co-medication and the number of co-medication is an independent association with ADEs due to antibiotic use [23], [29]. To prevent ADEs in elderly patients due to antibiotic use, the necessity of antibiotic treatment needs to be carefully determined, especially when co-medication is used. Minimizing unnecessary antibiotic treatment by even a small percentage could significantly reduce immediate and direct risks of ADE in individual patients [30].

The main strengths of this study are the long study period and the large representative study group. Since 1996 this RNH database is keeping records of all medications (including antibiotics) via a computerized medical registration program. Data accuracy can be guaranteed as data extraction takes place from electronic medical records of practices and regular training of the GPs and quality controls of the data take place [14], [16]. All patients with multiple antibiotic prescriptions per year have been included only once per year. Therefore, this study group is not biased by a few fragile elderly patients with multiple antibiotic prescriptions.

However, this study has several limitations. Firstly, antibiotic prescriptions could not be given in daily defined dosages (DDDs) like some international data [31], [32], limiting comparability with other studies. Secondly, we have no diagnostic information on acute infections. Thirdly, ADEs were self-reported by the patients, most probably underestimating the incidence of ADE associated with antibiotics.

Based on our findings, future strategies to decrease the antibiotic consumption and antibiotic resistance in the Netherlands should be addressed to all adult age categories. The elderly could be a specific target group and more in-depth study into the reasons for increasing antibiotic prescribing is necessary.
